# Effects of histamine and various histamine receptor antagonists on gene expression profiles of macrophages during compressive strain

**DOI:** 10.1007/s00056-021-00318-x

**Published:** 2021-07-06

**Authors:** Agnes Schröder, Catharina Petring, Anna Damanaki, Jonathan Jantsch, Peter Proff, Christian Kirschneck

**Affiliations:** 1 Department of Orthodontics, University Hospital Regensburg, Franz-Josef-Strauss-Allee 11, 93053 Regensburg, Germany; 2 Department of Periodontology and Operative Dentistry, University Medical Center of the Johannes Gutenberg University, Augustusplatz 2, 55131 Mainz, Germany; 3 Institute of Clinical Microbiology and Hygiene, University Hospital Regensburg, Franz-Josef-Strauß-Allee 11, 93053 Regensburg, Germany

**Keywords:** Osteoprotegerin, Orthodontic tooth movement, Compressive force, Cytokines, In vitro techniques, Osteoprotegerin, Kieferorthopädische Zahnbewegung, Druckbelastung, Zytokine, In-vitro-Techniken

## Abstract

**Purpose:**

Tissue hormone histamine can accumulate locally within the periodontal ligament via nutrition or may be released during allergic reactions by mast cells, which may have an impact on orthodontic tooth movement. In addition to periodontal ligament fibroblasts, cells of the immune system such as macrophages are exposed to compressive strain. The aim of this study was thus to investigate the impact of histamine on the gene expression profile of macrophages in the context of simulated orthodontic compressive strain.

**Methods:**

Macrophages were incubated with different histamine concentrations (50, 100, 200 µM) for 24 h and then either left untreated or compressed for another 4 h. To assess the role of different histamine receptors, we performed experiments with antagonists for histamine 1 receptor (cetirizine), histamine 2 receptor (ranitidine) and histamine 4 receptor (JNJ7777120) under control and pressure conditions. We tested for lactate dehydrogenase release and analyzed the expression of genes involved in inflammation and bone remodeling by reverse transcription quantitative polymerase chain reaction (RT-qPCR).

**Results:**

Histamine elevated gene expression of tumor necrosis factor under control conditions and in combination with pressure application. Increased prostaglandin-endoperoxide synthase‑2 mRNA was observed when histamine was combined with compressive force. Interleukin‑6 gene expression was not affected by histamine treatment. In macrophages, compressive strain increased osteoprotegerin gene expression. Histamine further elevated this effect. Most of the observed histamine effects were blocked by the histamine 1 receptor antagonist cetirizine.

**Conclusions:**

Histamine has an impact on the gene expression profile of macrophages during compressive strain in vitro, most likely having an impairing effect on orthodontic tooth movement by upregulation of osteoprotegerin expression.

## Abstract (de)

**Hintergrund:**

Das Gewebshormon Histamin kann sich lokal im parodontalen Ligament über die Ernährung anreichern oder bei allergischen Reaktionen von Mastzellen freigesetzt werden und dabei möglicherweise die kieferorthopädische Zahnbewegung beeinflussen. Neben parodontalen Ligamentfibroblasten sind auch Zellen des Immunsystems wie Makrophagen einer Druckbelastung ausgesetzt. Ziel dieser Studie war es daher, den Einfluss von Histamin auf das Genexpressionsprofil von Makrophagen im Kontext einer simulierten kieferorthopädischen Druckbelastung zu untersuchen.

**Methoden:**

Makrophagen wurden mit verschiedenen Histaminkonzentrationen (50, 100, 200 µM) für 24 h inkubiert und dann entweder unbehandelt gelassen oder für weitere 4 h komprimiert. Um die Rolle verschiedener Histaminrezeptoren zu beurteilen, führten wir Experimente durch mit Histamin-1- (Cetirizin), Histamin-2- (Ranitidin) und Histamin-4-Rezeptoren (JNJ7777120) unter Kontroll- und Druckbedingungen. Wir untersuchten die Freisetzung der Laktatdehydrogenase und analysierten die Expression an Entzündungsprozessen und am Knochenumbau beteiligten Genen mittels quantitativer Polymerasekettenreaktion („reverse transcription quantitative polymerase chain reaction“, RT-qPCR).

**Ergebnisse:**

Histamin erhöhte die Genexpression des Tumornekrosefaktors unter Kontrollbedingungen und in Kombination mit Druckapplikation. Bei Kombination von Histamin mit Druck wurde die Prostaglandin-Endoperoxid-Synthase‑2 verstärkt exprimiert. Die Interleukin-6-Genexpression wurde durch die Histaminbehandlung nicht beeinflusst. In Makrophagen erhöhte die Druckbelastung die Osteoprotegerin-Genexpression, dieser Effekt wurde durch Histamin noch weiter gesteigert. Die meisten der beobachteten Histamineffekte wurden durch den Histamin-1-Rezeptor-Antagonisten Cetirizin blockiert.

**Schlussfolgerungen:**

Histamin beeinflusst das Genexpressionsprofil von Makrophagen während Druckbelastung *in vitro* und beeinträchtigt durch die Hochregulierung der Osteoprotegerinexpression höchstwahrscheinlich die kieferorthopädische Zahnbewegung.

## Introduction

The dental discipline of orthodontics plays an important role for the prevention of oral diseases and supports oral health because anomalies in the deciduous or permanent dentition are reported to be predisposing risk factors for the development of gingivitis [39], chronic inflammatory processes in the periodontium [2, 39], and caries [10]. In orthodontics, a mechanical force is applied to the incorrectly positioned teeth by removable or fixed orthodontic appliances [11]. This mechanical force application causes the development of tensile and pressure zones in the periodontal ligament. In addition to fibroblasts, immune cells like macrophages are also exposed to mechanical strain during orthodontic tooth movement (OTM) [19, 23]. This mechanical stimulation triggers the secretion of proinflammatory enzymes, cytokines, and chemokines by periodontal ligament fibroblasts [15, 31, 44] as well as macrophages [32] and T cells [43]. Furthermore, chemotactic signaling substances attract monocytes and macrophages from the bloodstream, which contribute to the mediation of the sterile inflammatory reaction enabling OTM [18].

As a biogenic and vasoactive amine, histamine plays a key role in the modulation of allergic reactions [5]. In addition, histamine is a central proinflammatory mediator of inflammation in humans and other mammals influencing cell differentiation, proliferation and tissue homeostasis, while also being involved in cell regeneration [5]. As part of the innate immune response, histamine is released in the human body primarily by mast cells to defend against foreign substances, but it can also be ingested via nutrition. In many foods, histamine is produced in different concentrations by bacterial maturation and fermentation processes from the amino acid histidine [4, 42]. As an etiologic factor for food intolerance, histamine content is increasingly associated with symptoms such as diarrhea, headache, itching or flushing, referred to as so-called histamine intolerance [42]. In the human organism, histamine is generated by mast cells and released from intracellular vesicles as part of an immune reaction [5, 28]. This can lead to hypersensitive reactions of the skin including itching, reddening of the skin or edema, to vasoconstriction of the respiratory tract, to vasodilation with increased vascular permeability or even to an anaphylactic shock [5, 40].

The available literature lists four different histamine receptors, which differ in their function, structure, and occurrence in the human body as well as affinity for histamine [7, 40]. Depending on the stimulated cell type and histamine receptor subtype, histamine can therefore have a pro- and anti-inflammatory effect [40]. Histamine 1 receptors are present in numerous tissues and cells, including mast cells, and are involved in allergies and inflammation, mediating type 1 hypersensitivity reactions in addition to cell migration, vasodilation, and nociception [40]. The activation of histamine 2 receptors in immune cells, gastric mucosal cells, or smooth muscle cells has an impact on the permeability of vessels, induces gastric acid secretion, and stimulates other cells of the immune system [37, 40]. By regulating the release of various neurotransmitters, the histamine 3 receptor intervenes in the function of the blood–brain barrier and mediates neuroinflammatory diseases [38]. As this receptor is only expressed in neuronal tissues, we did not consider any histamine 3 receptor antagonists in this study. Like histamine 1 receptors, histamine 4 receptors are involved in the progression and modulation of allergies and inflammation, while controlling chemotactic immune-modulating processes via induced degranulation of mast cells [14, 21, 25, 30, 41]. Histamine receptor antagonists are taken by many patients in the context of allergic diseases. By specifically blocking the histamine 1 receptor, antagonists such as cetirizine mitigate allergic reactions in chronic urticaria [26] and allergic rhinitis [3] by impeding the release of inflammatory mediators and stabilizing mast cells [27]. Furthermore, histamine 4 receptor antagonists such as JNJ7777120 are becoming increasingly important in the treatment of mast-cell-mediated allergic diseases and also relieve inflammation and itching [40].

According to a health study by the German Robert Koch Institute, every fourth child or adult is diagnosed with an allergy in the course of their life, with hay fever being one of the most frequently reported allergy. Already 22.9% of all children and adolescents aged 0–17 years suffer from an allergic disease [9]. Based on these findings, it is reasonable to assume that some patients may suffer from allergic symptoms during orthodontic treatment and, as a result, frequently may take drugs to alleviate these allergies. Therefore, both histamine and histamine receptor antagonists released or taken during orthodontic treatment could impact OTM and thus the remodeling processes of the periodontium and the alveolar bone. The effects of simultaneous antihistamine therapy on OTM have so far been insufficiently investigated. To date, it is also not known to what extent cellular–molecular processes of macrophages during OTM are influenced and changed by the development of periodontal pressure zones and which role histamine plays in this situation. Therefore, we examined the gene expression profile of macrophages in the presence and absence of simulated orthodontic compressive strain under the influence of various histamine concentrations. In addition, we applied various histamine receptor antagonists to analyze which histamine receptor mediates histamine-induced effects in macrophages in the context of OTM.

## Materials and methods

### In vitro cell culture experiments

RAW264.7 macrophages (CLS Cell Lines Service, Eppelheim, Germany) were cultivated under standardized conditions (37 °C, 5% CO_2_) in Dulbecco’s Modified Eagle’s Medium—high glucose (DMEM, D5671, Sigma-Aldrich, St. Louis, MO, USA) with 10% fetal bovine serum (P30-3306, PAN-Biotech, Aidenbach, Germany); 1% L‑glutamine (G7513, Sigma-Aldrich, St. Louis, MO, USA) and 1% antibiotic/antimycotic solution (A5955, Sigma-Aldrich, St. Louis, MO, USA) in conventional T‑75 cell culture bottles (831.813.002, Sarstedt, Nümbrecht, Germany). All in vitro experiments took place on a sterile workbench (laminar flow unit, BDK air and cleanroom technology) and passages 16 to 32 were used.

To determine the most suitable concentration of histamine, 500,000 cells/ml of RAW264.7 macrophages were sown on 6‑well cell culture plates (353046, BD-Biosciences, San Jose, CA, USA) and 50, 100 or 200 µM histamine (H7125, Sigma-Aldrich, St. Louis, MO, USA) were added per well. In order to simulate mechanical–compressive strain, sterile glass plates of defined weight (2 g/cm^2^) were placed for 4 h after 24 h of preincubation [32].

To determine the role of different histamine receptors, RAW264.7 macrophages were treated with 50 µM histamine (H7125, Sigma-Aldrich, St. Louis, MO, USA) in combination with 100 µM histamine 1 receptor antagonist cetirizine (C3618, Sigma-Aldrich, St. Louis, MO, USA), 50 µM histamine 2 receptor antagonist ranitidine (R101, Sigma-Aldrich, St. Louis, MO, USA) or 100 µM histamine 4 receptor antagonist JNJ7777120 (J3770, Sigma-Aldrich, St. Louis, MO, USA) [12, 29].

### Assessment of cell number

Cell number was determined using a Coulter Particle Count and Size Analyser (Z2, Beckman Coulter, Brea, CA, USA). For this purpose, the cells were scrapped off in 1 ml of Dulbecco’s PBS (14190094, Life Technologies, Carlsbad, CA, USA). Then 100 μl of cell suspension was transferred to 10 ml of isotonic saline (0.8% NaCl in H_2_O_dd_) in a cell counter vessel (04-212-3000, Nerbe-Plus, Winsen/Luhe, Germany) and cell number was assessed.

### LDH cytotoxicity assay

A lactate dehydrogenase (LDH) test (4744926001, Sigma-Aldrich, St. Louis, MO, USA) was used to check the cytotoxic influence of individual stimuli, primarily of histamine and histamine antagonists under pressure. For this purpose, a LDH solution consisting of a catalyst and a dye solution was freshly prepared according to the manufacturer’s instructions. Each sample (50 μl) was mixed with 50 μl of the freshly prepared LDH solution and incubated in the dark at room temperature. The reaction was stopped after 30 min using a 25 μl stop solution in order to subsequently carry out measurements at 490 nm and at 690 nm in the ELISA reader (Multiscan GO Microplate Spectrophotometer, Thermo Fisher Scientific, Waltham, MA, USA).

### RNA isolation and cDNA synthesis

The RNA was isolated after the end of the incubation period with 500 µl peqGOLD TriFast (30-2010, VWR PEQLAB-Life Science, Darmstadt, Germany) per well. The RNA was further purified according to the manufacturer’s instructions. The RNA pellet obtained from these extraction steps was then resuspended in 20 μl of nuclease-free water (T143, Carl Roth, Karlsruhe, Germany). In order to quantify the amount of RNA, a photometric measurement of the optical density (OD) was performed at 280, 260, and 230 nm in the Nanodrop (NanoPhotometer® N60, Implen, Munich, Germany). A standardized amount of RNA with 0.1 nmol oligodT18 primer (SO131, Thermo Fisher Scientific, Waltham, MA, USA), 0.1 nmol random hexamer primer (SO142, Thermo Fisher Scientific, Waltham, MA, USA), 40 nmol dNTP (L785.2, Carl Roth, Karlsruhe, Germany), 1 × M-MLV buffer (M1705, Promega, Madison, WI, USA), 40 U RNase inhibitor (EO0381, Thermo Fisher Scientific, Waltham, MA, USA), 200 U reverse transcriptase (M1705, Promega, Madison, WI, USA) and nuclease-free water to a final volume of 20 µl were incubated for 60 min at 37 °C. The subsequent heating to 95 °C for 2 min then allowed the synthesized cDNA to be stored in a 1:10 dilution with nuclease-free water at −20 °C, since the reverse transcriptase was inactivated by this process. The complete RNA samples of an experiment were rewritten into cDNA at the same time to reduce experimental variations in the synthesis of the cDNA.

### Reverse transcription quantitative polymerase chain reaction (RT-qPCR)

Gene expression analysis was carried out using Mastercycler® ep realplex‑S thermocycler (Eppendorf, Hamburg, Germany) as described previously [31, 33, 35], whereupon 1.5 µl of the 1:10 diluted cDNA sample was mixed with a primer mix and complemented up to a total volume of 15 µl with nuclease-free water. The primer mix consisted of 7.5 µl SYBR®Green JumpStart^TM^ Taq ReadyMix^TM^ (S4438, Sigma-Aldrich, St. Louis, MO, USA) and 7.5 pmol of the respective primer pair (3.75 pmol/primer). All used primers were designed in accordance with the MIQE quality guidelines [6, 17]. In order to avoid possible contamination errors, a no-template control (NTC) without cDNA was carried out for each primer pair and for each qPCR. The cDNA amplification was carried out in duplicate using a RT-qPCR program with initial heat activation (95 °C, 5 min), followed by 45 cycles of denaturation (95 °C, 10 s), annealing (60 °C, 8 s), and extension (72 °C, 8 s). The fluorescence generated by the SYBR®Green was measured at a wavelength of 521 nm at the end of each cycle and Cq values were derived as the second derivate maximum of the fluorescence curve using the RealPlex software (version 2.2, Eppendorf, CalqPlex algorithm). To normalize target gene expression, the geometric Cq mean of two reference genes was used (Table 1), which proved to be stably expressed under the experimental conditions. Relative gene expression was calculated with the formula 2^-ΔCq^ with ΔCq = Cq (target gene)−Cq (geometric mean of reference genes) [17].

**Table 1:** RT-qPCR primer sequences for reference genes (*Eef1a1, Sdha*) and target genes RT-qPCR-Primersequenzen für Referenzgene (*Eef1a1, Sdha*) und Zielgene

Gene symbol	Gene name	5′-forward primer-3‘	5‘-reverse primer-3‘
*Eef1a1*	Eukaryotic translation elongation factor 1 α 1	AAAACATGATTACAGGCACATCCC	GCCCGTTCTTGGAGATACCAG
*Sdha*	Succinate dehydrogenase complex	AACACTGGAGGAAGCACACC	AGTAGGAGCGGATAGCAGGAG
*Ptgs-2*	Prostaglandin-endoperoxide synthase‑2	TCCCTGAAGCCGTACACATC	TCCCCAAAGATAGCATCTGGAC
*Il‑6*	Interleukin‑6	ACAAAGCCAGAGTCCTTCAGAG	GAGCATTGGAAATTGGGGTAGG
*Tnf*	Tumor necrosis factor	TCGAGTGACAAGCCTGTAGCC	CTTTGAGATCCATGCCGTTGGC
*Opg*	Osteoprotegerin	CCTTGCCCTGACCACTCTTAT	CACACACTCGGTTGTGGGT

### Statistical methods

Prior to statistics, absolute data values were divided by the arithmetic mean of the control group without mechanical strain to obtain normalized data values relative to these controls. Statistical analyses were calculated with GraphPad Prism 8.0 (GraphPad Software, San Diego, CA, USA). First, data were tested for normal distribution performing Shapiro–Wilk tests. Normally distributed datasets were independently compared by ordinary one-way analysis of variance (ANOVA), followed by Holm-Sidak’s multiple comparison tests, whereas the remaining datasets were compared by Welch-corrected ANOVAs, followed by Games–Howell’s multiple comparison tests. All differences were considered statistically significant at *p* ≤ 0.05.

## Results

### Impact of histamine concentrations on cell number and cytotoxicity

**Fig. 1:**
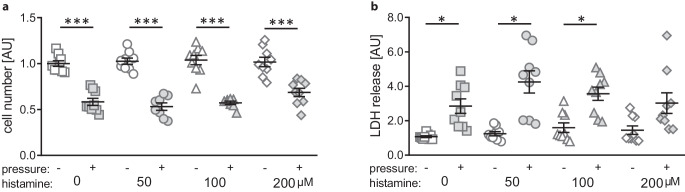
Determination of cell number (**a**) and cytotoxicity by lactate dehydrogenase (LDH) assay (**b**) after compressive force and histamine treatment. *AU* arbitrary units. *Horizontal lines *represent the arithmetic mean, *vertical lines* the standard error of the mean and *symbols* single data points. Statistics: cell number: ordinary analysis of variance (ANOVA) with Holm–Sidak’s multiple comparison test; LDH: Welch-corrected ANOVA with Games–Howell’s multiple comparison tests. *p ≤ 0.05; ***p ≤ 0.001 Bestimmung der Zellzahl (**a**) und der Zytotoxizität mittels LDH(Laktatdehydrogenase)-Assay (**b**) nach Druck- und Histaminbehandlung. *AU *Arbiträre Einheiten. *Horizontale Linien* stellen das arithmetische Mittel dar, *vertikale Linien* den Standardfehler des Mittelwertes und *Symbole* einzelne Datenpunkte. Statistik: Zellzahl: ANOVA („analysis of variance“) mit Holm-Sidak’s Post-hoc-Test; LDH: Welch-korrigierte ANOVA mit Games-Howell’s Post-hoc-Test. *p ≤ 0,05; ***p ≤ 0,001

First, we assessed the cell number (F = 32.6, df = 62, *p* < 0.001) and possible cytotoxic effects (W = 11.5; df = 25.44, *p* < 0.001) after 4 h of compressive force treatment and at least 28 h of histamine treatment. Mechanical strain significantly decreased cell numbers under control conditions (*p* < 0.001) and with all tested histamine concentrations (*p* < 0.001; Fig. 1a). Histamine itself had no effect on cell number in the tested concentrations without (50 µM: *p* = 0.998, 100 µM: *p* = 0.996, 200 µM: *p* = 0.998) and with additional pressure application (50 µM: *p* = 0.986, 100 µM: *p* = 0.998, 200 µM: *p* = 0.530). Mechanical strain increased LDH release within 4 h in macrophages without (*p* = 0.036) and with histamine treatment (50 µM: *p* = 0.020, 100 µM: *p* = 0.016), but not at the highest investigated concentration (200 µM: *p* = 0.321; Fig. 1b). According to cell number, we detected no cytotoxic effect of histamine without (50 µM: *p* = 0.891, 100 µM: *p* = 0.609, 200 µM: *p* = 0.803) and with compressive force treatment (50 µM: *p* = 0.618, 100 µM: *p* = 0.900, 200 µM: *p* > 0.999).

### Effects of histamine concentration on gene expression profile of macrophages

**Fig. 2:**
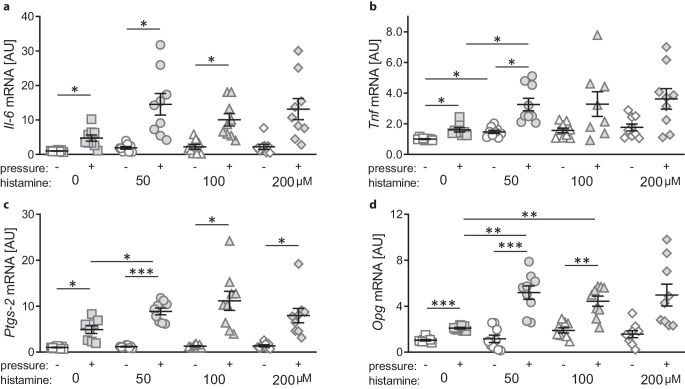
Gene expression of *Il‑6* (**a**), *Tnf* (**b**), *Ptgs‑2* (**c**) and *Opg* (**d**) after compressive force and histamine treatment. *AU* arbitrary units. *Horizontal lines* represent the arithmetic mean, *vertical lines* the standard error of the mean and *symbols* single data points. Statistics: Welch-corrected ANOVA (analysis of variance) with Games–Howell’s multiple comparison tests. *p ≤ 0.05; **p ≤ 0.01, ***p ≤ 0.001 Genexpression von *Il‑6* (**a**), *Tnf* (**b**), *Ptgs‑2* (**c**) und *Opg* (**d**) nach Druck- und Histaminbehandlung. *AU* Arbiträre Einheiten. *Horizontale Linien* stellen das arithmetische Mittel dar, *vertikale Linien* den Standardfehler des Mittelwertes und *Symbole* einzelne Datenpunkte. Statistik: Welch-korrigierte ANOVA („analysis of variance“) mit Games-Howell’s Post-hoc-Test. *p ≤ 0,05; **p ≤ 0,01, ***p ≤ 0,001

Next we analyzed the expression of proinflammatory genes like interleukin-6 (*Il‑6*; W = 10.63, df = 24.53, *p* < 0.001), tumor necrosis factor (*Tnf*; W = 13.11, df = 24.33; *p* < 0.001), and prostaglandin-endoperoxide synthase‑2 (*Ptgs‑2*; W = 21.99, df = 25.63, *p* < 0.001). Pressure application increased *Il‑6* gene expression under control (*p* = 0.040) conditions and with histamine treatment (50 µM: *p* = 0.046; 100 µM: *p* = 0.028; 200 µM: *p* = 0.085; Fig. 2a). Histamine had no effect on *Il‑6* gene expression under control conditions (50 µM: *p* = 0.328, 100 µM: *p* = 0.628, 200 µM: *p* = 0.717) or when combined with pressure application (50 µM: *p* = 0.155, 100 µM: *p* = 0.225, 200 µM: *p* = 0.255).

Like *Il‑6* gene expression, *Tnf* gene expression was elevated with the compressive force under control conditions (*p* = 0.027) and with 50 µM histamine (*p* = 0.028; Fig. 2b). With higher histamine concentrations, we no longer detected a significant increase of *Tnf* gene expression (100 µM: *p* = 0.484, 200 µM: *p* = 0.247). Addition of 50 µM histamine, enhanced *Tnf* gene expression without (*p* = 0.025) and with pressure application (*p* = 0.044), while higher concentrations failed to increase *Tnf* gene expression (without pressure: 100 µM: *p* = 0.083, 200 µM: *p* = 0.111; pressure: 100 µM: *p* = 0.502, 200 µM: *p* = 0.170; Fig. 2b).

As expected, *Ptgs‑2* gene expression was upregulated with compressive force treatment under all tested conditions (0 µM: *p* = 0.027, 50 µM: *p* < 0.001, 100 µM: *p* = 0.019, 200 µM: *p* = 0.036; Fig. 2c). Without pressure application histamine had no effect on the gene expression of *Ptgs‑2* at the tested concentrations (50 µM: *p* = 0.770, 100 µM: *p* = 0.488, 200 µM: *p* = 0.718). In contrast, *Ptgs‑2* gene expression was enhanced with 50 µM histamine (*p* = 0.045) combined with compressive force treatment, while higher histamine concentrations failed to increase *Ptgs‑2* gene expression (100 µM: *p* = 0.192, 200 µM: *p* = 0.670; Fig. 2c).

Next, we analyzed gene expression of osteoprotegerin (*Opg*; W = 27.79, df = 26.20, *p* < 0.001), which is involved in bone remodeling. Pressure application increased *Opg* gene expression under control conditions (*p* < 0.001) and with the addition of 50 and 100 µM of histamine (50 µM: *p* = 0.001; 100 µM: *p* = 0.005), whereas with 200 µM it only tended to increase *Opg* gene expression (*p* = 0.0865; Fig. 2d). Without mechanical loading, histamine had no effect on *Opg* gene expression (50 µM: *p* > 0.999, 100 µM: *p* = 0.065, 200 µM: *p* = 0.704). In contrast, 50 µM and 100 µM histamine further upregulated *Opg* expression during simultaneous compressive mechanical strain (50 µM: *p* = 0.001, 100 µM: *p* = 0.001; Fig. 2d).

### Effects of histamine receptor antagonists on cell number and cytotoxicity

**Fig. 3:**
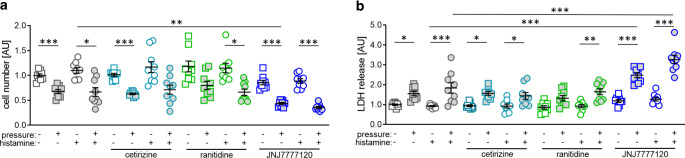
Determination of cell number (**a**) and cytotoxicity by lactate dehydrogenase (LDH) assay (**b**) after compressive force and treatment with 50 µM histamine with or without different histamine receptor antagonists. *AU* arbitrary units. *Horizontal lines* represent the arithmetic mean, *vertical lines* the standard error of the mean and *symbols* single data points. Statistics: cell number: Welch-corrected ANOVA (analysis of variance) with Games–Howell’s multiple comparison tests; LDH: ordinary ANOVA with Holm–Sidak’s multiple comparison test. *p ≤ 0.05; **p ≤ 0.01, ***p ≤ 0.001 Bestimmung der Zellzahl (**a**) und der Zytotoxizität mittels LDH(Laktatdehydrogenase)-Assay (**b**) nach Druckbelastung und Behandlung mit 50 µM Histamin mit oder ohne verschiedene Histaminrezeptorantagonisten. *AU* Arbiträre Einheiten. *Horizontale Linien* stellen das arithmetische Mittel dar, *vertikale Linien* den Standardfehler des Mittelwertes und *Symbole* einzelne Datenpunkte. Statistik: Zellzahl: Welch-korrigierte ANOVA („analysis of variance“) mit Games-Howell’s Post-hoc-Test; LDH: ANOVA mit Holm-Sidak’s Post-hoc-Test. *p ≤ 0,05; **p ≤ 0,01, ***p ≤ 0,001

As we detected most effects with 50 µM histamine, we decided to use this concentration for the experiments with histamine receptor antagonists. Again, we first analyzed the cell number (W = 43.03, df = 47.98, *p* < 0.001) and LDH release (F = 21.95, df = 47.99, *p* < 0.001). Pressure application was associated with reduced cell numbers for most tested conditions (Fig. 3a). However, remarkable differences between the untreated group and various histamine receptor antagonists became obvious. Without compressive force treatment, none of the tested inhibitors showed any effect on cell number without (cetirizine: *p* = 0.756, ranitidine: *p* = 0.739; JNJ7777120: *p* = 0.250) and with histamine addition (cetirizine: *p* = 0.999, ranitidine: *p* = 0.999; JNJ7777120: *p* = 0.127). This was also the case for cetirizine (control: *p* = 0.891, histamine: *p* > 0.999) and ranitidine (control: *p* = 0.871, histamine: *p* > 0.999) when combined with pressure application, while JNJ7777120 reduced cell number significantly if applied without histamine (*p* = 0.006; Fig. 3a).

As expected, LDH release was elevated after pressure application (*p* = 0.028; Fig. 3b). In line with cell number, we detected no cytotoxic effects by any of the tested antagonists without additional pressure application without (cetirizine: *p* = 0.992, ranitidine: *p* = 0.856; JNJ7777120: *p* = 0.577) or with the addition of 50 µM histamine (cetirizine: *p* = 0.998, ranitidine: *p* = 0.985; JNJ7777120: *p* = 0.297). Again, we detected no cytotoxic effects of cetirizine and ranitidine under compressive force treatment without (cetirizine: *p* = 0.998, ranitidine: *p* = 0.704) or with histamine (cetirizine: *p* = 0.180, ranitidine: *p* = 0.753). However, JNJ7777120 increased LDH release if applied with combined compressive force treatment without (*p* < 0.001) or with histamine supplementation (*p* < 0.001; Fig. 3b), indicating a toxic effect of JNJ7777120 upon pressure application.

### Effects of histamine receptor antagonists on gene expression profile of macrophages

**Fig. 4:**
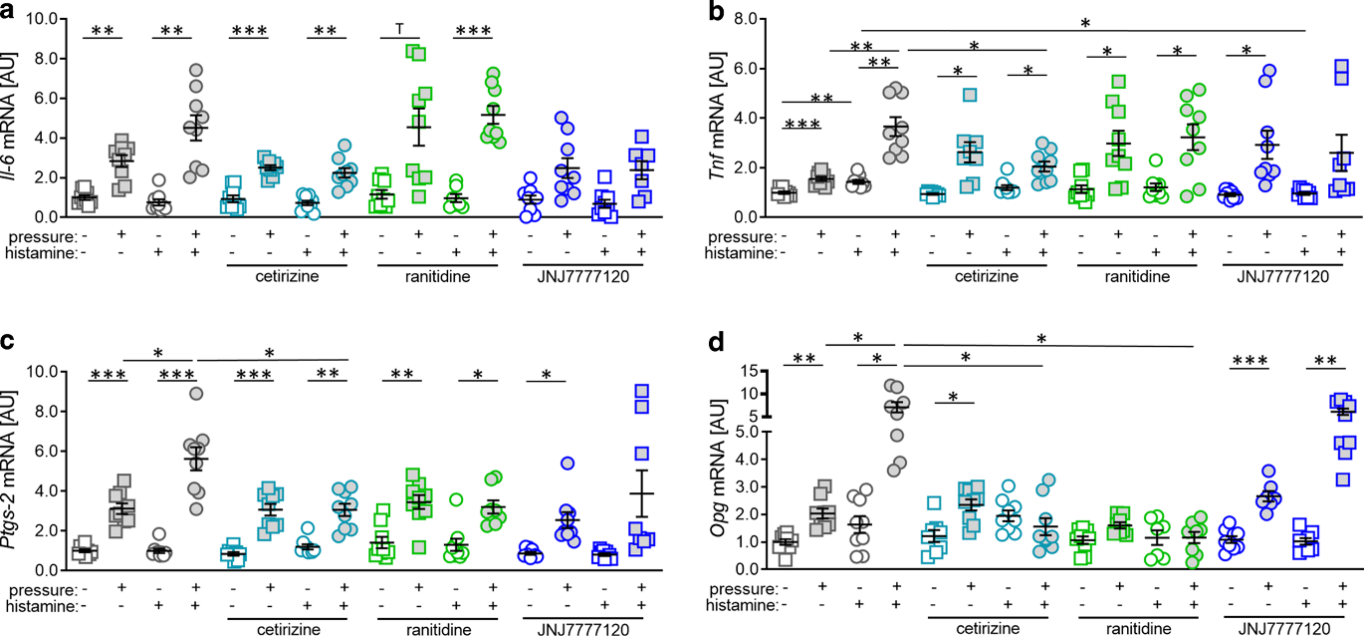
Gene expression of *Il‑6* (**a**), *Tnf* (**b**), *Ptgs‑2* (**c**) and *Opg* (**d**) after compressive force and treatment with different histamine receptor antagonists. *AU* arbitrary units. *Horizontal lines* represent the arithmetic mean, *vertical lines* the standard error of the mean and *symbols* single data points. Statistics: Welch-corrected ANOVA (analysis of variance) with Games–Howell’s multiple comparison tests; *p ≤ 0.05; **p ≤ 0.01, ***p ≤ 0.001 Genexpression von *Il‑6* (**a**), *Tnf* (**b**), *Ptgs‑2* (**c**) und *Opg* (**d**) nach Druckbelastung und Behandlung mit verschiedenen Histaminrezeptorantagonisten. *AU* Arbiträre Einheiten. *Horizontale Linien* stellen das arithmetische Mittel dar, *vertikale Linien* den Standardfehler des Mittelwertes und *Symbole* einzelne Datenpunkte. Statistik: Welch-korrigierte ANOVA („analysis of variance“) mit Games-Howell’s Post-hoc-Test; *p ≤ 0,05; **p ≤ 0,01, ***p ≤ 0,001

Next, we investigated the effects of the different histamine receptor antagonists on proinflammatory genes to evaluate, which receptor subtype mediating the histamine-induced effects is important in the context of OTM. Pressure-induced *Il‑6* gene expression (W = 17.67, df = 46.15, *p* < 0.001) was not affected by the tested antagonists if applied without (cetirizine: *p* = 0.913, ranitidine: *p* = 0.799; JNJ7777120: *p* = 0.913) or with parallel histamine treatment (cetirizine: *p* > 0.999, ranitidine: *p* = 0.799; JNJ7777120: *p* > 0.999; Fig. 4a), which was not surprising, as we neither detected a histamine effect on *Il‑6* gene expression under control (*p* = 0.668) nor pressure conditions (*p* = 0.164; Fig. 4a).

In contrast, *Tnf* gene expression (W = 12.04, df = 47.22, *p* < 0.001) was increased with histamine under control conditions (*p* = 0.008) and with mechanical loading (*p* = 0.007; Fig. 4b). Combined with cetirizine we still detected pressure-induced *Tnf* gene expression (control: *p* = 0.050, histamine: *p* = 0.039), but the histamine effect was significantly reduced when combined with pressure application (*p* = 0.041; Fig. 4b). This effect was not observed with ranitidine (*p* = 0.057), while JNJ7777120 abolished the histamine effect under control conditions (*p* = 0.004), but did not significantly impact on the histamine effect during mechanical strain (*p* = 0.886; Fig. 4b).

Gene expression of *Ptgs‑2* (W = 19.25, df = 47.07, *p* < 0.001) was only affected, when histamine was combined with compressive force treatment (*p* = 0.033; Fig. 4c). Neither cetirizine (*p* = 0.925), ranitidine (*p* = 0.974), nor JNJ7777120 (*p* = 0.814) affected gene expression of *Ptgs‑2* without simultaneous pressure application. Only cetirizine reduced *Ptgs‑2* gene expression during mechanical strain combined with additional histamine significantly (*p* = 0.031; Fig. 4c), while the other antagonists had no effect (ranitidine: *p* = 0.974, JNJ7777120: *p* = 0.866).

Finally, we tested the effects of various histamine receptor antagonists on the gene expression of *Opg *(W = 11.09, df = 44.33, *p* < 0.001; Fig. 4d). We detected a histamine effect in combination with mechanical strain (*p* = 0.033). This effect was truncated with cetirizine (*p* = 0.020) and ranitidine treatment (*p* = 0.014; Fig. 4d), whereas JNJ7777120 had no significant effect on *Opg* expression during a combined mechanical compressive strain and histamine treatment (*p* = 0.993).

## Discussion

Macrophages can modify the extent of orthodontic tooth movement (OTM) by regulating the release of cytokines and chemokines [13, 32]. Therefore, the influence of histamine and mechanical force application on macrophages was examined in this work. Histamine had no effect on the number of macrophages or on cytotoxicity, whereas static pressure application had a cell-number-reducing and cytotoxic effect. In addition, under control conditions without mechanical treatment, histamine only marginally increased the expression of proinflammatory genes in macrophages, whereas static compression significantly stimulated the expression of *Ptgs‑2, Il‑6* and *Tnf*. *Opg* expression was promoted by histamine and by static compression. We determined the histamine 1 receptor as the most important histamine-effect-mediating receptor in macrophages, as incubation with the histamine 1 receptor antagonist cetirizine led to a truncation of the histamine-induced effects.

While 50 µM histamine were most effective in macrophages, 100 µM histamine were identified to be necessary to have an impact on the expression of proinflammatory factors in periodontal ligament cells in a recent study [12], indicating a higher sensibility of macrophages to histamine. In the study by Mommert et al., bone-marrow-derived macrophages were treated with different histamine concentrations ranging from 0.1 to 100 µM to determine effects of histamine on gene expression of oncostatin M [24]. This study investigated a broad range of histamine concentrations in macrophages and justified this with different affinities and expression levels of the histamine 1, histamine 2 and histamine 4 receptors [24]. However, the concentration used in this study corresponded to the results of Czerner et al., who also described a concentration-dependent effect of histamine with a maximum for 50 µM histamine in macrophages [8].

Effects of orthodontic forces on macrophages were already described by Alhashimi et al. [1] and by He et al. [13]. Alhasimi et al. examined the expression of costimulatory molecules at orthodontically treated teeth in an animal model and analyzed the expression of CD40 by macrophages at the resorption as well as the tension side of the treated rat molars showing an immune-modulating response by macrophages after mechanical force induction [1]. The study by He et al. showed that the mechanical force induced accumulation of M1-like macrophages, while the synthesis of proinflammatory mediators such as TNF induced alveolar bone resorption and ultimately tooth movement [13].

Compressive force application increased gene expression of *Ptgs‑2, Il‑6*, and *Tnf* in macrophages. From this, we concluded that an increased inflammatory response also occurs in macrophages during OTM. It is known from literature that macrophages participate in physiological and nonphysiological processes of immunity, inflammation, and tissue remodeling. Therefore, an involvement in bone-remodeling processes is obvious [16].

We detected no gene expression of receptor activator of NF-κB ligand (*Rankl*) in macrophages, while gene expression of RANKL decoy receptor *Opg* was elevated with compressive strain in macrophages. This reaction was contrary to that of periodontal ligament fibroblasts, which reduce *Opg* gene and protein expression due to mechanical strain [31, 35]. However, it was already reported that *Opg* expression is, among others, controlled by the transcription factor NFAT‑5 (nuclear factor of activated T‑cells), which thereby has an osteoprotective function [36]. In recent studies, it has been shown that NFAT‑5 is stabilized in macrophages due to mechanical strain and sodium chloride in the extracellular medium [32, 34]. In chondrocytes, histamine showed an impact on the RANKL/OPG ratio by modulating RANKL expression with only slight effects on OPG expression [22]. This was in line with our finding that histamine did not have an impact on *Opg* mRNA levels under control conditions. Treatment with histamine in combination with mechanical stress increased *Opg* expression in macrophages, indicating a bone-protective mechanism and effect of histamine on macrophages during OTM. Histamine was furthermore reported to be directly involved in osteoclast differentiation through autocrine and paracrine action on osteoclast precursor cells and through an increased RANKL/OPG expression ratio in osteoblasts [4]. Macrophages express histamine 1, histamine 2, and histamine 4 receptors, but not the histamine 3 receptor [25]. Mommert et al. examined gene expression and functions of various histamine receptors in monocytes and fully differentiated M1 macrophages with a focus on the histamine 4 receptor. They determined new immunomodulatory functions of histamine receptor type 4 in this cell type [25]. In our hands, histamine 4 receptor antagonist JNJ7777120 in combination with mechanical strain had cytotoxic effects on macrophages. This contradicts the results of Czerner et al., who showed significant antagonization of histamine-induced effects applying JNJ7777120 in RAW267.4 macrophages [8]. Other studies also used the antagonist JNJ7777120 as a well-usable inhibitor of histamine 4 receptors in their experiments [20, 21]. Czerner et al. furthermore reported that RAW264.7 cells predominantly expressed histamine 1 and histamine 4 receptors and only bone marrow-derived macrophages expressed histamine 2 receptors slightly [8]. This could be an explanation for the nonsignificant inhibitory effect of the histamine 2 receptor antagonist ranitidine on the stimulating histamine effects in RAW264.7 macrophages.

## Conclusions

- Expression of histamine 1, histamine 2 and histamine 4 receptors enables macrophages to detect histamine concentrations in the periodontal tissue surrounding the teeth.
- Increased histamine concentrations seem to be accompanied by a slightly increased expression of inflammatory cytokines. A distinct upregulation of osteoprotegerin expression in mechanically strained macrophages can have an impairing effect on osteoclastogenesis and orthodontic tooth movement in vivo.
- Since cetirizine significantly reduced the proinflammatory and bone-protective gene expression response of macrophages, this effect appears to be mainly mediated by the histamine 1 receptor.
